# Strong correlation between urine and vaginal swab samples for bacterial vaginosis

**DOI:** 10.4102/sajid.v36i1.199

**Published:** 2021-06-17

**Authors:** Deshanta Naicker, Veron Ramsuran, Meleshni Naicker, Fazana Dessai, Jennifer Giandhari, Partson Tinarwo, Nathlee Abbai

**Affiliations:** 1 School of Clinical Medicine Research Laboratory, Nelson R Mandela School of Medicine, University of KwaZulu-Natal, Durban, South Africa; 2 KwaZulu-Natal Research and Innovation Sequencing Platform (KRISP), School of Laboratory Medicine and Medical Sciences, University of KwaZulu-Natal, Durban, South Africa; 3 Centre for the AIDS Programme of Research in South Africa (CAPRISA), University of KwaZulu-Natal, Durban, South Africa; 4 Department of Biostatistics, Nelson R Mandela School of Medicine, University of KwaZulu-Natal, Durban, South Africa

**Keywords:** pregnant women, bacterial vaginosis, urine, swab, BV, ddPCR, *G. vaginalis*, South Africa

## Abstract

**Background:**

Vaginal swabs have been traditionally used for the diagnosis of bacterial vaginosis (BV). Currently, there are limited studies that have investigated the use of other sample types other than vaginal swabs for the detection of BV from South African populations. This study investigated whether urine can be used for the detection of BV-associated microorganisms in South African pregnant women.

**Methods:**

One-hundred self-collected vaginal swabs and urine samples were obtained from women presenting for antenatal care at King Edward VIII Hospital in Durban. The BD MAX™ vaginal panel assay was used for diagnosing BV and droplet digital polymerase chain reaction was used to quantify *Gardnerella vaginalis, Prevotella bivia, Atopobium vaginae* and *Lactobacillus crispatus*. The absolute counts were determined on the QX200 Droplet Reader (Bio-Rad) using the QuantaSoft Software. Data analysis was performed with statistical computing software called R, version 3.6.1.

**Results:**

Median copy numbers obtained for *G. vaginalis* and *P. bivia* across urine and swabs in BV-positive samples were not significantly different (*p* = 0.134 and *p* = 0.652, respectively). This was confirmed by the correlation analysis that showed a good correlation between the two sample types (*G. vaginalis* [*r* = 0.63] and *P. bivia* [*r* = 0.50]). However, the data obtained for *A. vaginae* differed, and a weak correlation between urine and swabs was observed (*r* = 0.21). Bacterial vaginosis-negative samples had no significant difference in median copy numbers for *L. crispatus* across the urine and swabs (*p* = 0.062), and a good correlation between the sample types was noted (*r* = 0.71).

**Conclusion:**

This study highlights the appropriateness of urine for the detection of microorganisms associated with BV.

## Background

Bacterial vaginosis (BV) is a clinical condition that is caused by alterations of the vaginal microbiota. Predominant, normal lactobacilli species are replaced by diverse communities of anaerobic and facultative bacteria.^1,2,3^ These bacteria include: *Gardnerella vaginalis, Atopobium vaginae, Prevotella, Mycoplasma* and numerous other microorganisms.^2,3,4,5,6^

The prevalence of BV in women has been associated with socio-demographic factors, diagnostic criteria, gestational age, vitamin D deficiency and smoking.^7,8^ South Africa has the highest prevalence of BV in Africa.^9^ The prevalence of BV was reported to be 17.6% in a population of pregnant women in the Gauteng province.^9^ A more recent study conducted by our research group reported a prevalence of 49.4% for BV in antenatal women from Durban.^10^

The common symptom of BV is the occurrence of an abnormal malodorous (strong fishy odour) vaginal discharge.^3^ Implications of untreated BV include pelvic inflammatory disease (PID) and an increased susceptibility to sexually transmitted infections (STIs) and human immunodeficiency virus (HIV).^11,12,13^

Several reports indicate that BV-positive women have a higher incidence of HIV infection. Furthermore, an increased severity of BV is associated with increased prevalence of HIV.^13,14,15,16^ Untreated BV is associated with severe pregnancy outcomes which includes late miscarriages, preterm labour, premature rupture of membranes (PROMs), post-partum endometritis, low birth weight infants and a host of other complications. Bacterial vaginosis therefore proves to be a major health risk in pregnancy.^6,17,18^

Bacterial vaginosis is classically diagnosed in the laboratory using the Nugent Scoring System as a gold standard.^5,6,14,18,19^ Other methods of diagnosing BV include the use of Amsel Criteria and nucleic acid amplification tests.^3,5,6,18^ Current diagnostic methods require the use of vaginal swabs to determine BV status. Collection of swabs by a clinician using a speculum to detect BV can be an uncomfortable and invasive method of sample collection, especially during pregnancy. Therefore, this method of collection may not be ideal for pregnant women. Although self-collected vaginal swabs are not considered invasive, it can pose a level of discomfort for the women during collection. In contrast, urine is a non-invasive sample. In majority of the primary healthcare clinic settings, pregnant women are screened for glucose and leukocytes by providing a urine sample to be tested.^20^ Therefore, obtaining urine as a sample type used to test BV is far more attainable and easier.

This study compared the bacterial load of BV-associated microorganisms in paired urine and self-collected vaginal swab samples. The microorganisms investigated in this study included: *Gardnerella vaginalis (G. vaginalis), Atopobium vaginae (A. vaginae), Prevotella bivia (P. bivia)* and *Lactobacillus crispatus (L. crispatus). Gardnerella vaginalis, A. vaginae* and *P. bivia* were selected because these microorganisms are most associated with a positive diagnosis for BV. *Lactobacillus crispatus* was selected as this microorganism is associated with a healthy vaginal microbiota (dominant in BV-negative women). This study provides the first report on correlation between urine and vaginal swabs for the detection and the quantification of BV-associated microorganisms in South African pregnant women.

## Methods

### Study setting and population

In this study, 273 pregnant women 18 years and older, willing to provide written informed consent as well as swab and urine samples, were included in the analysis. The women were recruited from the King Edward VIII Hospital in Durban, South Africa, during November 2017 – April 2018. Each woman provided three self-collected vaginal swabs (collected before the urine sample) and thereafter a urine sample (not restricted to first void only). The women were provided with instructions on how to collect the vaginal swab samples. The study population included both symptomatic women as well as asymptomatic women. Women who reported symptoms were treated as per the syndromic management guidelines.^21^

### Laboratory testing

#### Study design and sample selection

This study was a sub-study of a larger study which recruited 273 pregnant women. Samples were tested using the BD MAX^TM^ vaginal panel and stratified into BV-positive and BV-negative. The final subset for this study contained matched urine and swab pairs for 25 BV-positive and 25 BV-negative women. These 100 samples were further tested using droplet digital polymerase chain reaction (ddPCR) technology.

#### Diagnosis of bacterial vaginosis

The BV status of the enrolled women was determined using the BD MAX™ Vaginal Panel assay (Becton Dickinson) on the self-collected swabs as per the manufacturer’s instructions. The women were diagnosed as either BV-positive or BV-negative.

#### Deoxyribonucleic acid isolation from swabs and urine

The extraction of the swab and urine samples was performed using the PureLink Microbiome deoxyribonucleic acid (DNA) purification kit (Invitrogen, Cat No. A29790, ThermoFisher Scientific [Waltham, Massachusetts, United States]). For the extraction of the urine, a starting volume of 10 mL of sample was used. The sample was centrifuged, and the extraction was performed on the cell pellet. The vaginal swab was immersed in 1 mL of phosphate buffered saline (PBS) and gently vortexed to dislodge the sample material from the swab. After vortexing, the swab was discarded and the PBS containing the vaginal material was used for the extraction. Similarly, to the urine extraction, the sample was centrifuged, and the extraction was performed on the cell pellet. The extractions were performed as per the manufacturer’s instructions.

#### Droplet digital polymerase chain reaction

Detection and quantification of the investigated microorganisms were performed by ddPCRthat provides absolute quantification of target genes. For the ddPCR assay, predesigned TaqMan primer and probes (ThermoFisher Scientific) were used to quantify *G. vaginalis* (Assay ID: Ba04646236_s1), *P. bivia* (Assay ID: Ba04646278_s1), *A. vaginae* (Assay ID: Ba04646222_s1) and *L. crispatus* (Assay ID: Ba04646245_s1). A total of 2.5 µL of extracted DNA from urine and swabs was used in a 20-µL ddPCR reactions with the 2× digital PCR supermix for probes (No dUTP). Droplets were generated using the manual droplet generator, Droplet Generation Oil for Probes (Bio-Rad, Hercules, CA, USA) and the PCR mix containing the sample. A total of 40 µL of droplets were used for the PCR reaction, with the following conditions: 95 °C for 10 min, 40 cycles of 94 °C for 30 s and 60 °C for 1 min and 98 °C for 10 min. Cycled PCR reactions were read on the QX200 Droplet Reader (Bio-Rad, Hercules, CA, USA) using the QuantaSoft Software and acquired on channel 1 for 6-carboxyfluorescein (FAM). Analysis was performed on the QuantaSoft Software using manual thresholding.

#### Statistical analysis

Descriptive analysis and inferential statistics were conducted. Wilcoxon’s non-parametric test was used to compare any differences in measurements between the groups. The distributions within the groups were also visually displayed as boxplots. The association between the binary outcome (BV status) and the categorical demographic profile was assessed using the chi-square test or Fisher’s exact test in the case of smaller frequencies. All the analysis was performed with the aid of a freely available Statistical Computing software called R, version 3.6.1. The correlation between urine and vaginal swab groups was evaluated using Spearman’s correlation (GraphPad, USA) and significance was tested at *p* < 0.05. For Spearman’s correlation, *R* > 0.7 is considered a strong correlation, *R* value between 0.5 and 0.7 considered a moderate correlation and *R* < 0.4 is considered a weak correlation.

### Ethical considerations

Full ethics approval for the present study was granted by the Biomedical Research Ethics Committee (BREC) of the University of KwaZulu-Natal (BE276/18).

## Results

### Characteristics of study population

In this population, a higher proportion of the women did not experience symptoms of abnormal vaginal discharge at enrolment (66% vs. 34%) (Table 1). Most of the women had attained a high school level of education (64%) and were unmarried (84%). With respect to sexual behaviour, most women had reported having a regular sexual partner (90%), experiencing sexual debut between the ages of 15–20 years (84%), having between two and four lifetime sexual partners (46%) and not practicing condom use during their last sex act (66%). Most women were in the third trimester of pregnancy (56%) and had no previous history of STIs (52%). When stratified according to BV status, trimester of pregnancy was found to have a statistically significant association with the BV status (*p* = 0.04). A higher proportion of BV-negative women (72%) was observed in the third trimester, whilst higher proportion of BV-positive women (24% and 36%, respectively) was observed in the first and second trimesters of pregnancy (Table 1). That is, women in the third trimester were 91% less likely to be BV-positive when compared to those in the first trimester (*p* = 0.039). However, there was not enough evidence to suggest a significant difference in the likelihood of being BV-positive between the women in the first and second trimesters of pregnancy (*p* = 0.249) (data not shown).

**TABLE 1:** Characteristics of the women investigated in this study.

Variable	BV positive		BV negative		Total		*p*
	*n*	%	*n*	%	*n*	%	
**Total**	**25**		**25**		**50**		**-**
**Age†,‡**							0.602
**Current abnormal discharge**							0.136
No	14	56	19	76	33	66	-
Yes	11	44	6	24	17	34	-
**Level of education**							0.377
Primary school	1	4	0	0	1	2	-
High school	14	56	18	72	32	64	-
College or university	10	40	7	28	17	34	-
**Married**							0.702
No	22	88	20	80	42	84	-
Yes	3	12	5	20	8	16	-
**Has a regular sex partner**							0.050*
No	0	0	5	20	5	10	-
Yes	25	100	20	80	45	90	-
**Cohabiting with partner**							0.390
No	13	52	16	64	29	58	-
Yes	12	48	9	36	21	42	-
**Age of first sex**							0.830
< 15	1	4	1	4	2	4	-
15–20	22	88	20	80	42	84	-
21–25	2	8	3	12	5	10	-
c25	0	0	1	4	1	2	-
**Number of lifetime sex partners**							0.816
1	6	24	7	28	13	26	-
2–4	11	44	12	48	23	46	-
> 4	8	32	6	24	14	28	-
**Partner has other partners**							0.186
No	5	20	9	36	14	28	-
Yes	12	48	6	24	18	36	-
Do not know	8	32	10	40	18	36	-
**Condom use**							0.597
Never	6	24	5	20	11	22	-
Rarely	3	12	1	4	4	8	-
Sometimes	15	60	16	64	31	62	-
Always	1	4	3	12	4	8	-
**Condom use at last sex act**							0.370
No	15	60	18	72	33	66	-
Yes	10	40	7	28	17	34	-
**Intravaginal practices**							0.110
Yes	4	16	0	0	4	8	-
No	21	84	25	100	46	92	-
**Trimester of pregnancy**							0.040
1st	6	24	1	4	7	14	-
2nd	9	36	6	24	15	30	-
3rd	10	40	18	72	28	56	-
**Past preterm delivery**							0.189
No	20	80	23	95.8	43	87.8	-
Yes	5	20	1	4.2	6	12.2	-
**Past miscarriage**							0.480
No	19	76	21	84	40	80	-
Yes	6	24	4	16	10	20	-
**Abnormal discharge in the past**							0.254
No	12	48	16	64	28	56	-
Yes	13	52	9	36	22	44	-
**Previous treatment of sexually transmitted infections**							1.000
No	13	52	13	52	26	52	-
Yes	12	48	12	48	24	48	-

BV, bacterial vaginosis; SD, standard deviation.

* , Statistical significance, *p ≤* 0.05.

† , Mean: BV-positive = 29.2; BV-negative = 28.4; Total = 28.8;

‡ , SD: BV-positive = 5.0; BV-negative = 6.7; Total = 5.9.

## Laboratory findings

### Comparison of deoxyribonucleic acid readings in urine and swab samples according to bacterial vaginosis status

**FIGURE 1:**
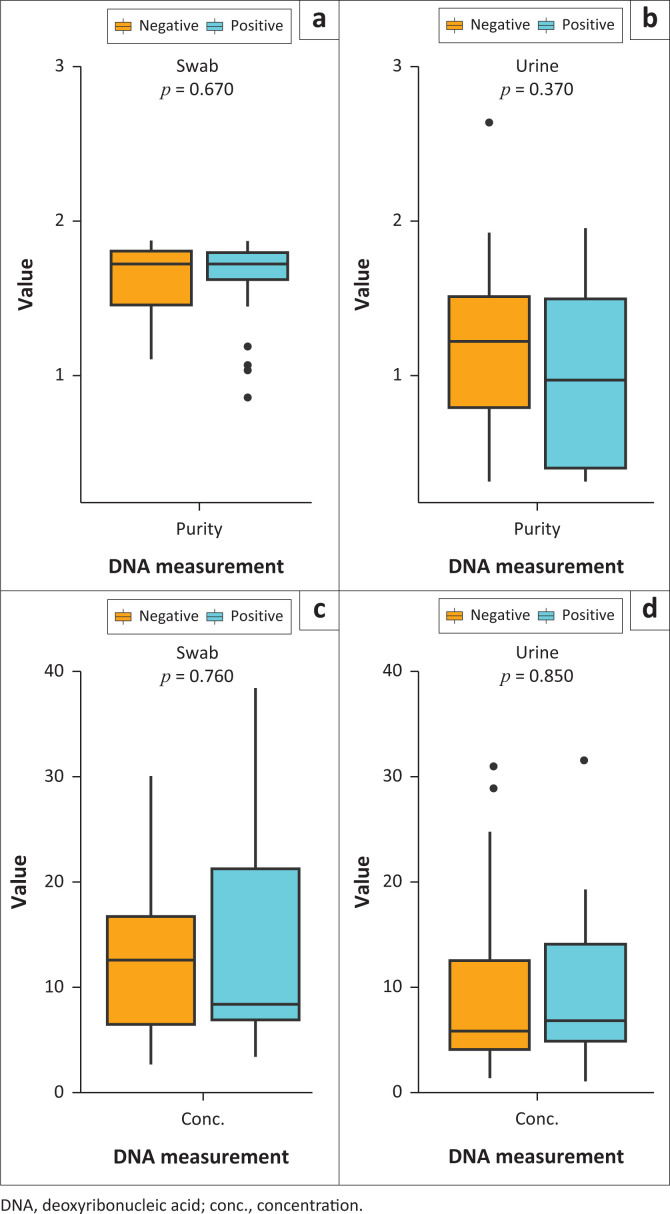
Comparison of the deoxyribonucleic acid purity and concentration values in the swab and urine samples across the bacterial vaginosis states. There was no significant difference (*p* > 0.05) in deoxyribonucleic acid purity and concentration values in urine and swab samples across bacterial vaginosis-positive and bacterial vaginosis-negative samples. (a) DNA purity of swab samples, (b) DNA purity of urine samples, (c) DNA concentration of swab samples, (d) DNA concentration of urine samples.

When comparing the median DNA concentration and purity values in the urine and the swab samples across BV-positive and BV-negative samples, there was no significant difference (*p* > 0.05) (Figure 1 and Appendix 1). This indicates that there was no bias in the quality or integrity of the different sample types used for the ddPCR reactions.

### Comparison of the abundance of each bacterium in bacterial vaginosis-negative and bacterial vaginosis-positive women

**FIGURE 2:**
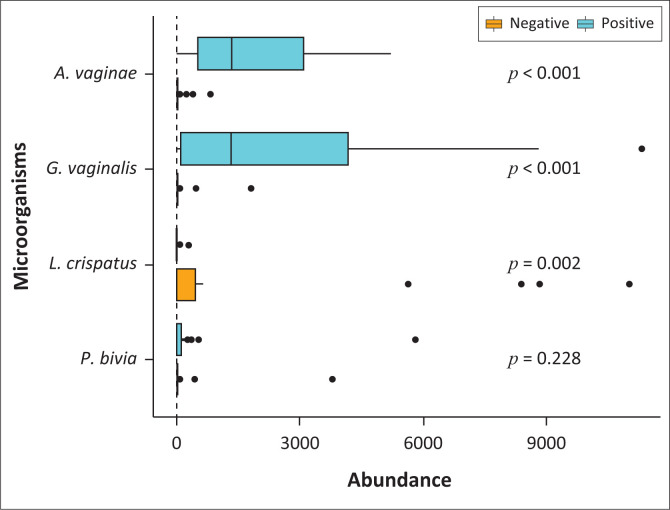
The abundance of each microorganism in relation to bacterial vaginosis status. *Gactobacillus vaginalis* was shown to be the most abundant microorganism in bacterial vaginosis-positive samples. *Lactobacillus crispatus* was the most dominant microorganism in bacterial vaginosis-negative samples.

*Gardnerella vaginalis* was observed as the most abundant microorganism, followed by *A. vaginae* and *P. bivia* in the BV-positive women, whereas *L. crispatus* was the most abundant microorganism in the BV-negative women. As expected, a higher abundance of *A. vaginae* and *G. vaginalis* was observed in BV-positive samples when compared to BV-negative samples. The level of abundance of these microorganisms between the two BV states was found to be statistically significant (*p* < 0.001). However, there was no statistical significance between the abundance of *P. bivia* across the BV states (*p* = 0.228). In keeping with previous reports, a higher abundance of *L. crispatus* was shown to be present in the BV-negative samples when compared to the BV-positive samples (*p* = 0.002) (Figure 2).

### Comparison of the abundance of each microorganism in urine versus swab samples

**FIGURE 3:**
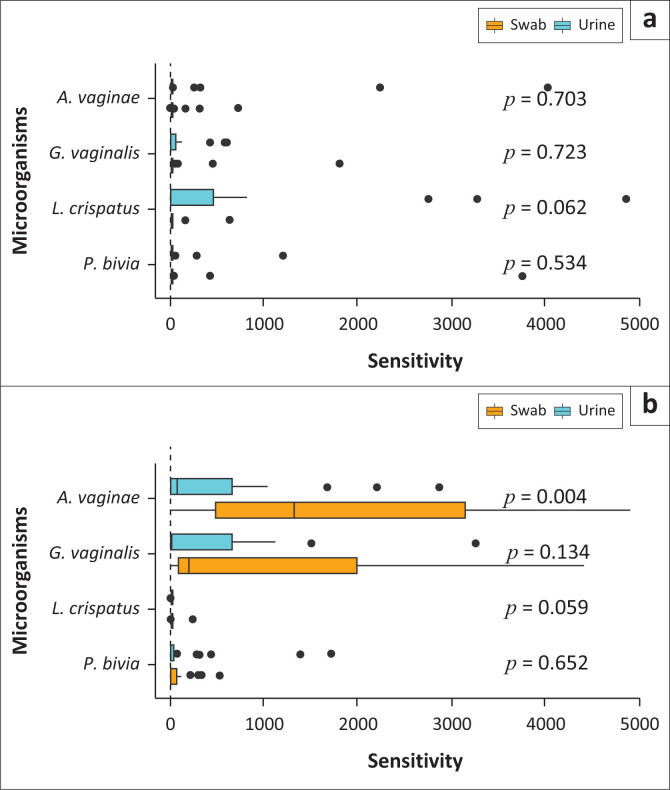
Comparison of the abundance of each microorganism in bacterial vaginosis-positive and bacterial vaginosis-negative groups in urine versus swab samples: (a) negative and (b) positive.

The median copy numbers of each microorganism quantified from urine were compared to the copy numbers of each microorganism quantified from the swab samples. This comparison was made across the BV-positive and BV-negative samples. According to Figure 3 for the BV-negative samples, there were no significant differences in the median copy number of each microorganism between the urine and swab samples (*p* > 0.05). However, in the BV-positive samples, a higher median copy for *A. vaginae* was observed in the swab samples when compared to the urine samples (*p* = 0.004).

**FIGURE 4:**
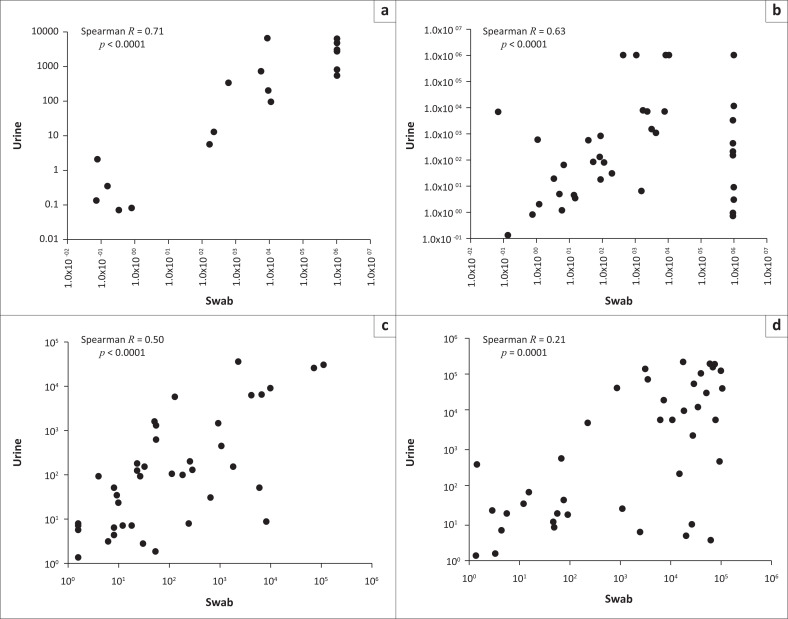
The graph on the top left shows the correlation of urine versus swab samples for the detection and quantification of *Lactobacillus crispatus*. A strong correlation between the sample types, *R* = 0.71, *p* < 0.0001, was observed. The plot on the top right shows the correlation of urine versus swab samples for *Gardnerella vaginalis*. A moderate correlation between urine and swab samples, *R* = 0.63, *p* < 0.0001, was observed. The graph on the bottom left displays the correlation of urine versus swab samples for *Prevotella bivia* as shown here. A moderate correlation between urine and swab samples, *R* = 0.50, *p* < 0.0001, was observed, followed by the correlation of urine versus swab samples for *Atopobium vaginae*. A weak correlation between urine and swab samples, *R* = 0.21, *p* = 0.001, was observed. (a) *Lactobacillus crispatus,* (b) *Gardnerella vaginalis,* (c) *Prevotella bivia and* (d) *Atopobium vaginae.*

A Spearman’s correlation for the swab and urine samples for each microorganism was performed. A strong correlation between the two sample types was noted for *G. vaginalis, P. bivia* and *L. crispatus* (Figure 4). These data are in accordance with Figure 3 that showed that there is no significant difference in the copy numbers across both sample types for the microorganisms above. However, for *A. vaginae*, a weak correlation between urine and swab samples was noted (Figure 4). This was expected, because there was a significant difference in the microbial load between urine and swab samples for this microorganism (Figure 3).

## Discussion

This study provides the first evidence for the detection and quantification of BV-associated bacteria (*G. vaginalis, P. bivia, A. vaginae* and *L. crispatus*) in urine samples collected from South African pregnant women.

This study showed *G. vaginalis* to be the most abundant microorganism in the BV-positive samples followed by *A. vaginae* and *P. bivia* (Figure 2). This correlates with previous studies that have reported *G. vaginalis* to be the leading microorganism associated with BV.^3,22,23,24^ The levels of abundance of *G. vaginalis* are used as the indicator for diagnosis of BV.^24^ In the current study, a higher microbial load of *G. vaginalis* was detected in the urine and swab samples of BV-positive women when compared to the BV-negative women. In addition, the findings from the present study indicated that urine is an appropriate sample for detection and quantification of *G. vaginalis* because a good correlation was obtained between the urine and swabs (*R* = 0.63, *p* < 0.0001). These findings are supported by other published studies. In a study conducted by Swidsinski et al.,^25^ the authors reported on the presence of *G. vaginalis* in first-void urine samples obtained from German pregnant women. More recently, Datcu et al.^20^ also demonstrated the potential to detect *G. vaginalis* from a urine sample collected from women in the general population of Greenland.

In this study, *A. vaginae* was the second most abundant microorganism detected amongst the BV-associated bacteria. A higher abundance of *A. vaginae* was shown to be present in the urine and swab samples of BV-positive women when compared to BV-negative women. This was expected as *Atopobium* species have been detected in the vaginal microbiota of women with BV.^26^ A study conducted by Bradshaw et al.^27^ also reported on the presence of *A. vaginae* in Australian women diagnosed with BV in Melbourne, Australia.

In the current study for the BV-positive women, a higher median copy for *A. vaginae* was observed in the swab samples when compared to the urine samples (*p* = 0.004). This resulted in a poor correlation (*R* = 0.21, *p* = 0.001) between the two sample types.

*Prevotella* was reported as the least abundant microorganism quantified in this study. Previous studies have reported a lower abundance of *Prevotella* when compared to *G. vaginalis* in BV-positive women.^5,28^ The current study found a moderate correlation (*R* = 0.50, *p* ≤ 0.0001) between the urine and swabs samples for the detection of *P. bivia.* Similar results were found in a study conducted by Datcu et al.,^20^ which showed a low level of detection of *Prevotella* from urine, thereby confirming our study findings. After an extensive survey of the literature, there were no published findings on the detection of *A. vaginae* and *P. bivia* from urine samples collected from pregnant women, thereby limiting the discussion of the current study. However, the current study now provides data that fill this gap in knowledge.

According to our analysis, *L. crispatus* was shown to be present at a higher abundance in the BV-negative women when compared to the BV-positive women. This result was expected as *L. crispatus* is associated with a healthy vaginal microbiota because *L. crispatus* produces lactic acid and other compounds that are inhibitors of bacterial species that are positively associated with BV.^2,29,30^ Obtaining data on the abundance of bacteria that are positively and negatively associated with BV will assist with future studies that aim to determine the bacterial load cut-off in a urine sample. This provided the rationale for the inclusion of *L. crispatus* in the current study. In this study, *L. crispatus* was shown to be present in both urine and swabs samples from women who were classified as BV-negative. A good correlation between the two sample types was observed (*R* = 0.71, *p* < 0.0001).

The present study had the following limitations: we did not attempt to investigate every known vaginal microorganism known to cause BV (BV-associated bacteria [BVAB 1,2,3], *Mobiluncus*) because of funding constraints; the gold standard Nugent scoring method was not used for the diagnosis of BV because we found that this technique did not work well with self-collected vaginal swabs (inadequate sample material on the majority of the swabs thereby making the microscopic evaluations challenging), and lastly, the present study was a pilot study that only included 100 patient samples.

However, the strengths of this study are as follows: the stratification according to BV status was performed using an FDA-approved automated assay for the diagnosis of BV (i.e. BD MAX™ Vaginal panel assay), which is extremely sensitive and specific, as well as superior to microscopy which is highly subjective. Despite the small sample size, the study was able to show significant correlations between the urine and swabs for the detection and quantification of the investigated microorganisms.

## Conclusion

The results obtained in this study indicate the successful detection and quantification of all investigative BV-associated microorganisms from urine samples using ddPCR. The detection of BV-associated microorganisms from urine offers a much more comfortable and less-biased sampling method when compared to vaginal swabs (bias because downstream processing is dependent on amount of material on the swab). The lack of published data on the detection of BV-associated microorganisms from urine samples collected from pregnant women in the South African and African setting lends novelty to the present study. In addition, there are no published works on the use of ddPCR for absolute quantification of BV-associated microorganisms. The data from this study can be used as preliminary data to develop larger studies which investigate the feasibility of this technology for future diagnostics.

## Appendix 1

### Supplementary data

**TABLE 1-A1:** Median value for the deoxyribonucleic acid purity and concentration values in swab and urine samples across the bacterial vaginosis (BV) states.

BV status	BV negative	*N* = 25	BV positive	*N* = 24	*p*	Overall	*N* = 49
**Swab samples**							
Purity	-	-	-	-	0.652	-	-
Median (Q1–Q3)	1.71	1.46–1.81	1.70	1.61–1.82	Ranksum	1.71	1.47–1.81
Min–Max	1.11–1.87	-	0.880–1.87	-	-	0.880–1.87	-
Concentration	-	-	-	-	0.734	-	-
Median (Q1–Q3)	13.3	6.90–19.4	11.9	7.08–27.4	Ranksum	13.3	7.00–21.3
Min–Max	2.60–76.4	-	3.50–135	-	-	2.60–135	-
**Urine samples**							
Purity	-	-	-	-	0.342	-	-
Median (Q1–Q3)	1.22	0.770–1.48	0.965	0.418–1.46	Ranksum	1.11	0.660–1.48
Min–Max	0.320–26.6	-	0.320–1.97	-	-	0.320–26.6	-
Concentration	-	-	-	-	0.834	-	-
Median (Q1–Q3)	8.80	4.70–14.2	8.60	4.88–16.1	Ranksum	8.80	4.80–15.5
Min–Max	1.65–521	-	1.20–585	-	-	1.20–585	-

**TABLE 2-A1:** Median values for the abundance of bacterial vaginosis-associated microorganisms across the bacterial vaginosis (BV) states.

BV status	BV negative	*N* = 25	BV positive	*N* = 25	*p*	Overall	*N* = 50
***G. vaginalis***							
Swab concentration	-	-	-	-	< 0.001	-	-
Median (Q1–Q3)	3.50	0.0700–37.2	8700	1080–1 000 000	Ranksum	106	1.85–753 000
Min–Max	0–1 000 000	-	0.0700–1 000 000	-	-	0–1 000 000	-
Urine concentration	-	-	-	-	0.002	-	-
Median (Q1–Q3)	3.40	0.190–125	1130	18.3–10 900	Ranksum	74.0	1.40–6210
Min–Max	0–1 000 000	-	0–1 000 000	-	-	0–1 000 000	-
***P. bivia***							
Swab concentration	-	-	-	-	0.228	-	-
Median (Q1–Q3)	0.600	0.150–6.70	2.70	0.170–94.0	Ranksum	1.25	0.155–13.9
Min–Max	0–3750	-	0–5800	-	-	0–5800	-
Urine concentration	-	-	-	-	0.327	-	-
Median (Q1–Q3)	1.20	0.290–7.60	2.60	0.400–31.1	Ranksum	2.10	0.320–18.8
Min–Max	0–1210	-	0–1710	-	-	0–1710	-
***A. vaginae***							
Swab concentration	-	-	-	-	< 0.001	-	-
Median (Q1–Q3)	0.620	0.0700–4.50	1340	527–3120	Ranksum	88.4	0.253–1330
Min–Max	0–738	-	0.0700–5230	-	-	0–5230	-
Urine concentration	-	-	-	-	0.002	-	-
Median (Q1–Q3)	1.00	0.0800–11.1	544	1.30–5600	Ranksum	7.35	0.318–1510
Min–Max	0–7220	-	0–1 000 000	-	-	0–1 000 000	-
***L. crispatus***							
Swab concentration	-	-	-	-	< 0.001	-	-
Median (Q1–Q3)	0.350	0–11 000	0	0–0	Ranksum	0	0–119
Min–Max	0–1 000 000	-	0–227	-	-	0–1 000 000	-
Urine concentration	-	-	-	-	0.001	-	-
Median (Q1–Q3)	0.360	0.0700–737	0	0–0.0900	Ranksum	0.0800	0–4.65
Min–Max	0–6620	-	0–13.6	-	-	0–6620	-
